# The Multi-Elemental Composition of the Aqueous Humor of Patients Undergoing Cataract Surgery, Suffering from Coexisting Diabetes, Hypertension, or Diabetic Retinopathy

**DOI:** 10.3390/ijms22179413

**Published:** 2021-08-30

**Authors:** Jolanta Flieger, Joanna Dolar-Szczasny, Robert Rejdak, Dariusz Majerek, Małgorzata Tatarczak-Michalewska, Jędrzej Proch, Eliza Blicharska, Wojciech Flieger, Jacek Baj, Przemysław Niedzielski

**Affiliations:** 1 Department of Analytical Chemistry, Medical University of Lublin, Chodźki 4A, 20-093 Lublin, Poland; malgorzatatatarczakmichalewska@umlub.pl (M.T.-M.); eliza.blicharska@umlub.pl (E.B.); 2 Department of General and Pediatric Ophthalmology, Medical University of Lublin, Chmielna 1, 20-079 Lublin, Poland; joannaszczasny@op.pl (J.D.-S.); robert.rejdak@umlub.pl (R.R.); 3 Department of Applied Mathematics, University of Technology, Nadbystrzycka 38D, 20-618 Lublin, Poland; majerek@gmail.com; 4 Faculty of Chemistry, Department of Analytical Chemistry, Adam Mickiewicz University in Poznań, 89B Umultowska Street, 61-614 Poznań, Poland; jed.proch@gmail.com (J.P.); pnied@amu.edu.pl (P.N.); 5 Department of Anatomy, Medical University of Lublin, 20-090 Lublin, Poland; wwoj24@umgmail.com (W.F.); jacek.baj@umlub.pl (J.B.)

**Keywords:** elemental analysis, aqueous humor (AH), cataract, diabetes, hypertension, diabetic retinopathy, age-related macular degeneration (AMD), ICP-OES

## Abstract

The aim of the study was the multi-elemental analysis of aqueous humor (AH) collected from patients undergoing cataract surgery. The study included: 16 patients with age-related macular degeneration AMD (99 controls), 10 patients with retinopathy (105 controls), 61 patients with hypertension (54 controls), and 33 patients with coexisting diabetes (82 controls). The control groups were recruited from patients with a lack of co-existing disease characterizing the specified studied group. The measurements were performed by the use of inductively coupled plasma optical emission spectrometry (ICP-OES). The statistical analysis was carried out using non-parametric testing (Mann–Whitney U). The level of significance was set at *p* = 0.05. The data obtained revealed substantial variations in elemental composition between the test groups in comparison to the controls. However, the significant variations concerned only a few elements. The phosphorous (P) level and the ratio of P/Ca were significant in retinopathy and diabetes, whereas cobalt (0.091 ± 0.107 mg/L vs. 0.031 ± 0.075 mg/L; *p* = 0.004) was significant in AMD. In co-existing hypertension, the levels of tin (0.293 ± 0.409 mg/L vs. 0.152 ± 0.3 mg/L; *p* = 0.031), titanium (0.096 ± 0.059 mg/L vs. 0.152 ± 0.192 mg/L; *p* = 0.045), and ruthenium (0.035 ± 0.109 mg/L vs. 0.002 ± 0.007 mg/L; *p* = 0.006) varied in comparison to the controls. The study revealed inter-elemental interactions. The correlation matrices demonstrated the domination of the positive correlations, whereas negative correlations mainly concerned sodium.

## 1. Introduction

Many adults experience significant vision loss because of their elderly age or common ocular diseases such as age-related macular degeneration (AMD), cataracts, diabetic retinopathy (DR), or glaucoma.

Despite available treatments, their etiology still remains poorly understood. Therefore, there is a need to search for factors influencing eye health as well as uncover efficient prevention strategies.

A cataract is a progressive loss of transparency in the eye’s lens, accounting for almost 50% of blindness in the world [1]. So far, the mechanism of cataractogenesis has not been fully understood. However, several factors increasing the risk of cataract formation have been identified, namely cigarette smoking, genetic predisposition, oxidative stress, exposure to UV light, systemic diseases such as diabetes, hypertension, or homocystinuria, drugs’ intake, for example, steroids, and co-existing eye diseases such as retinitis pigmentosa or uveitis [2,3,4]. Biochemical analysis of the eye tissues appears to be a useful diagnostic tool enabling the identification of the causes of many dysfunctions. The lens and AH of living patients are usually collected during routine cataract surgery. AH is of great importance to the normal functioning of the eye, as it satisfies the metabolic needs of the lens and the other intraocular structures. The fluid from an anterior chamber volume is about 0.3–0.5 mL. AH is produced constantly, owing to an active secretion by the non-pigmented ciliary epithelium [5,6]. This is probably a more predominant source of eye fluid than diffusion and ultrafiltration of the plasma. In fact, it demonstrated that the difference in the concentration of various ions (for example, HCO_3_^−^, and Cl^−^) in comparison to blood plasma appears to be much larger than might be expected from the Gibbs–Donnan equilibrium. Moreover, AH exhibits differences in protein composition in comparison with plasma.

Chowdhury et al. [7] identified more than six hundred proteins in AH by nanoflow liquid chromatography electrospray ionization tandem mass spectrometry (nano-LC-ESI-MS/MS), and antibody protein arrays. Proteomic analysis revealed the existence of proteins with catalytic, enzymatic, and structural properties. Most of these were described for the first time. The authors established the concentrations of identified cytokines, chemokines, and receptors that ranged between 0.1 and 2.5 ng mL^−1^. AH was also tested for, among others, oxidative–antioxidant balance. The relationship between cataract maturity and markers of lipid peroxidation and activity of enzymatic antioxidants such as superoxide dismutase (SOD) and catalase (CAT) was assessed. Multiple logistic regression analyses identified them as predictors of cataract maturity [8]. It was established that ascorbic acid and glutathione (GSH), considered the main antioxidants protecting the eye against oxidative stress, are present in AH on the level of 1 mM and 2 μM, respectively [9,10]. The gas chromatography combined with time-of-flight mass spectrometry (GC-TOF-MS) of AH revealed some key regulatory elements referred to amino acids and lipids metabolisms such as glutaric acid and pelargonic acid in relation to cataract progression [11]. It was also proven that the 25-hydroxyvitamin D (25 (OH) D) level in AH is statistically associated (*p* = 0.006) with diabetic cataracts [12]. Tryptophan (TRP) and its catabolites: kynurenic acid (KYNA) and kynurenine (KYN) in AH were examined using ultra-high-performance liquid chromatography coupled to electrospray ionization triple quadrupole mass spectrometry (UHPLC-ESI-MS/MS) in fluid from the anterior chamber of the eye from cataract patients [13]. The obtained results suggest that neuroactive metabolites of TRP in the kynurenine pathway may play a significant role in cataract formation in patients suffering from diabetes. Flieger et al. [13] proved the increased level of KYNA and KYN, and the ratio of TRP/KYN in AH of cataract patients suffering from diabetes as compared to controls.

Besides components such as immunoglobulins, neuropeptides, proteins, lipids, carbohydrates, enzymes, the vitamins, AH was also examined for its elemental composition. It is known that electrolyte balance is of vital importance to lens transparency. Inorganic ions move through ion channels such as potassium, sodium, chloride, and calcium channels relying on ion exchange (Na^+^/H^+^, Na^+^/Ca^2+^, HCO_3_^−^/Cl^−^) or active transport thanks to Na-K-ATPase [14]. The relationship between the abnormal increase in sodium concentration, or the impaired functional activity of Na-K-ATPase, and the opacification of the human lens cortex has been confirmed [14]. Magnesium deficiency, accompanied by the impairment of ATPase, may also lead to the development of cataracts [15,16].

The first study on the presence of heavy metals in ocular tissue was carried out by Zeimer et al. [17]. Later on, Erie et al. [18] performed an analysis of AH obtained postmortem to determine its elemental composition using inductively coupled plasma–mass spectrometry (ICP-MS). In 2014, [19] TXRF (total reflection X-ray fluorescence spectrometry) analysis of the lens and AH of cataract patients showed a higher level of chromium and manganese in both media, an elevated concentration of barium in the lens, and nickel in AH. The authors suggested further study to elucidate ocular metal accumulation. Dolar-Szczasny et al. [20] studied alterations of chemical element levels in the AH of cataract patients by inductively coupled plasma optical emission spectrometry (ICP-OES). The obtained results illustrate the significant diversity of the concentration levels of elements in the AH. The detected elements were divided by hierarchical cluster analysis into five independent groups depending on the variation in the levels of elements in the studied population. The cluster with elements found at the highest concentration levels contained Ca, Cs, K, Mg, Na, P, and Rb. The authors emphasized that the AH samples of patients with cataracts were characterized by elevated levels of very toxic elements such as thallium (Tl), tellurium (Te), cesium (Cs), lead (Pb), or aluminum (Al). It is known that the toxic metals act through interactions with the specific proteins, resulting in a change in their structure, and hence their function [21]. Melanin in retinal pigment epithelium melanosomes is such a protein taking part in the binding of essential and toxic heavy metals. Several reports [18,22,23] have proven that the retina and choroid can accumulate lead and cadmium as a result of chronic exposure to these toxic elements. However, the above study failed to confirm the presence of other metals such as mercury and thallium in the fluids and tissues of the eyes.

In accordance with the literature data, cataracts occur more frequently and at an earlier age in diabetic patients rather than in non-diabetic ones [24,25]. The presence of diabetes accelerates cataract formation mainly by inducing the glycation of lens proteins by glucose [26]. Fe, Zn, and Cu contents of AH, lens, and serum samples of nondiabetic patients and diabetic patients were analyzed by atomic absorption spectrometry (AAS) [21]. It appeared that the levels of the investigated elements in AH and serum of the diabetics in comparison to non-diabetic patients were not different. However, the lens levels of Cu in diabetic patients were significantly higher as compared with nondiabetic patients (*p* = 0.02). In 2019, Kayiklik et al. [26] examined the content of selected elements such as Ca, P, Mg, Na, K of AH in diabetic and non-diabetic patients with cataracts as for the occurrence of pseudoexfoliation (PSX). The authors, having applied photometric quantification, observed low Na levels in the anterior chamber of patients with pseudoexfoliation (PSX) and posterior subcapsular cataracts (PSCC), a high P level in diabetic non-PSX eyes, and high Ca and Cl levels in the non-diabetic PSX (+) group.

Stopa et al. [27] examined levels of AH trace elements in patients with AMD. The authors’ findings concerning iron (Fe), cadmium (Cd), and copper (Cu) support these elements’ role as environmental risk factors for AMD. However, the authors declared higher levels of zinc in the AH of patients with AMD, which contrasts with other studies. The observed accumulation of iron is also considered as a risk factor in AMD, mainly due to the generation of free radicals through the Fenton reaction.

Hohberger Bettina et al. [28] established levels of aqueous humor trace elements in patients with open-angle glaucoma. The authors observed that patients with primary open-angle glaucoma (POAG) and pseudoexfoliation glaucoma (PEXG) presented with significantly higher AH levels of zinc in comparison to controls. This finding supports the previous hypothesis that these trace elements are involved in the pathogenesis of open-angle glaucoma. Additionally, instead of the single-element levels, the authors also used element/element ratios as markers for the real element status.

The levels of macro-and micronutrients change dynamically in the human body, and it is indisputable that these are of critical importance to human health. Many studies concern the determination of the level of both essential and toxic metals in different tissues, taken from patients suffering from various diseases, including eye problems. However, the results of these studies appear problematic, chiefly due to the variety of methods used (AAS, ICP-MS, ICP-AES, AAS, LA-ICP-MS). The inconsistency of the results obtained by different teams be due to the criteria of selecting the test and control groups or the unquestionable inter-patient variability.

Taking into account the individual variability of patients and the inability to collect a control group composed only of healthy volunteers, the research findings still need to be verified on new and larger groups of patients inhabiting different environments and suffering from varied coexisting diseases. The aim of the current study was to evaluate the elemental composition of AH in patients undergoing cataract surgery, additionally suffering from diabetes, hypertension, or diabetic retinopathy. The control group consisted of patients undergoing cataract surgery without distinguished co-morbidities. Statistical analysis of the results will allow for the hypothesis to be verified regarding the existence of differences (or their lack) in the composition of the eye fluid of cataract patients between the selected subgroups.

## 2. Results and Discussion

### 2.1. Elemental Analysis

In the present study, AH samples were examined by using ICP-OES with the aim to measure the concentration of 67 elements that were divided into 5 clusters according to the previous report [20]. All measured values were tested statistically using non-parametric testing (Mann–Whitney U). The level of significance was set at *p* = 0.05. Table S1 shows the mean concentrations for all studied elements in the AH of cataract patients suffering from hypertension, diabetes, diabetic retinopathy, and age-related macular degeneration (AMD).

Many toxic metals have been found in examined samples such as mercury (Hg), thallium (Tl), cadmium (Cd), aluminum (Al), or lead (Pb). Pb was found in all samples studied at the range from 0.56 to 1.66 ppm, Al was in the range from 1.03 to 2.59 ppm, and Cd occurred at a much lower concentration, from 0 to 0.013 ppm. AH toxic metals’ concentrations reflect an exposure of all patients to these metals, which are known to be permanent industrial pollutants standing behind the environmental contamination. Despite the fact that there are many pieces of evidence indicating that these metals can cause oxidative stress by ROS exert production, resulting, among others, in lipid peroxidation, DNA damage, and the depletion of cellular antioxidant defense systems [29,30,31], no statistically significant correlations were found, proving the role of these as differentiating factors for the studied patients’ groups.

The highest concentration range was observed for Ca, Cs, K, Mg, Na, P, and Rb. Table 1 lists the average concentrations of these elements belonging to cluster number 5 (previous classification [20]) along with control-corrected values and the standard variations. The large standard deviations observed are not surprising considering the diversity of patients.

As can be seen, there are no significant differences in the AH levels of the most abundant elements in comparison to the control groups for each patients’ group (*p* > 0.05). Only the concentration of phosphorous (P) in the AH of patients suffering from diabetes (*p* = 0.017) and diabetic retinopathy (*p* = 0.012) was significantly smaller in comparison to appropriate controls.

### 2.2. The Inter-Elemental Correlations

**Figure 1:**
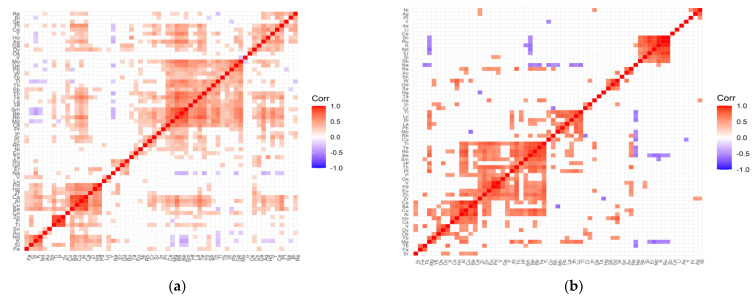
Correlation matrix for the control (**a**) and test (**b**) groups representing patients with cataract and coexisting AMD.

**Figure 2:**
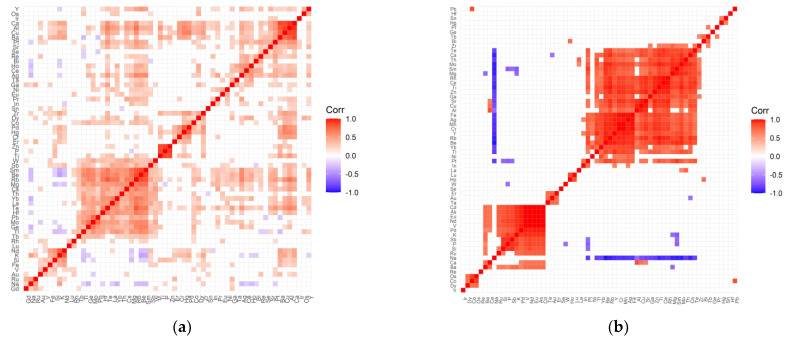
Correlation matrix for the control (**a**) and test (**b**) groups representing patients with cataract and coexisting retinopathy.

**Figure 3:**
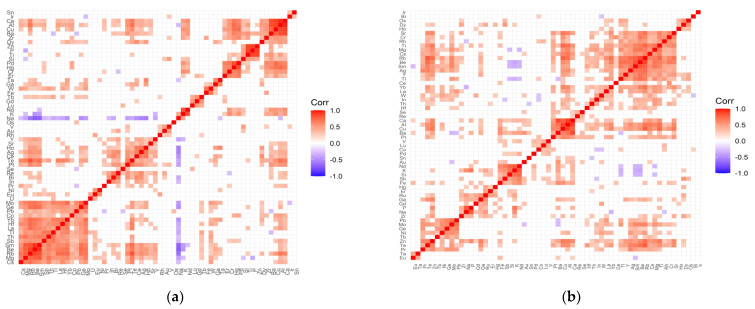
Correlation matrix for the control (**a**) and test (**b**) groups representing patients with cataract and coexisting hypertension.

**Figure 4:**
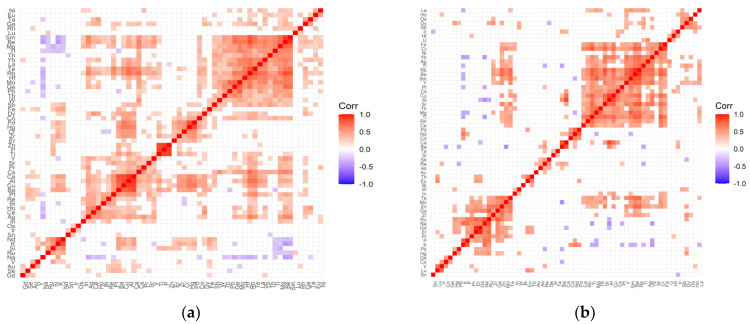
Correlation matrix for the control (**a**) and test (**b**) groups representing patients with cataract and coexisting diabetes.

Variable cleanup was performed for all data. Elements that showed no variation were removed from both the test and control groups. Then, a correlation analysis was performed for each tested group of patients and the respective controls. Figure 1, Figure 2, Figure 3 and Figure 4 present matrices obtained for cataract patient groups with coexisting AMD, hypertension, diabetes, and retinopathy. On the correlation matrices, the formation of clusters of the elements correlating with each other are observed. Due to the strength of the correlation between the elements, there were red fields for positively correlating variables, blue fields for negative correlations, and white fields for no significant correlation.

In the case of the control group for AMD (Figure 1a), the following clusters of strongly, positively correlated elements are visible: (Mg, Ge, Pb, W, Tb, Tl, Th, Sb, Yb, Te, La, Hf, Sm, Be, Rb, Mg, Cs); (Pd, Hg, Zr, Ca, Al, Cu, Ba, Co); (Zn, P, Ti—very strong correlations); (Nd, K, Si, Fe); (Pt, Ce, Y, Ho, Ag, Ga, Dy). On the other hand, in the case of the study group for AMD (Figure 1b), the following clusters can be distinguished: (Ti, Te, Rb, Be, Sm, Hf, Th, Pt, P, Ge, V, Pd, Eu, Zn, Zr); (Tl, Lu, Bi, La, Pb, Mo, Rh, Co); (Ru, K, Nd, Er, Si, Sb); (W, Gd, Se); (Ca, Ba, Cu, Al, Ha, Cs); (Ir, Dy, Os); (Re, Au); (Tb, Fe). The strength of negative correlations for the control group is quite weak, while the AMD group shows an increase in negative correlations, which mainly concern sodium negatively correlated with Tl, Lu, Bi, Mo, Rh, Co, Te, Rb, Se, Tb, Mg, and magnesium correlated with Sc, Ru, K, Nd, Er. In addition, there are single negative correlations of the following pairs of elements: Mo/Ba, Hg/Zr, Cr/Rh, Y/Co, K/Sm.

The following positively correlated clusters can be seen in the correlation matrix for the retinopathy control group (Figure 2a): (Ca, Al, Cu, Ba, Pt, Sr, Se); (Re, Bi, Ho, Ce, Ag); (Zr, Dy, Co, Pd, Hg, Cr, Er); (Zn, P, Ti—strong correlations); (W, Sb, Sm, Be, Rb, Mg, Cs, Th, Yb, La, Te, Hf, Pb, Mo, Ge, Tl, Tb); (Nd, K, Si, Fe); (Ru, Na, Gd). In the case of the studied group (Figure 2b), the correlation matrix contains much more distinct clusters: (Te, Cs, Th, Mo, Sm, Mg, Rh, Ce, Ti, Zn, Ga, Sr, Cu, Al, Fe, Ag, Cr, Y, Rb, Be, Yb, Tl); (Pt, In); (Lu, Ho); (Er, Au, Ta); (Nd, Eu, V, Pd, K, Sb, P, Si, Ru); (Os, Co, Dy). The negative correlations for the control group are very weak, while for the test group with retinopathy, there are strong negative correlations that relate to sodium correlated with Te, Cs, Th, Mo, Sm, Mg, Rh, Ce, Ti, Zn, Ga, Sr, Cu, Al, Fe, Ag, Mn, Cr, Y, Rb, Be, Yb, Tl, Pt. In addition, in the study group, there are single negative correlations of the following pairs of elements: Mg/Ba, W/P, Cs/Au, Mg/K, Pt/(Sb, P, Si), Sm/(K, Sb, P).

The following positive correlations can be distinguished in the correlation matrix of the control group for hypertension (Figure 3a): (Mo, Ge, Pb, Co, Yb, Hf, La, Tl, Th, Sb, Sm, Be, Rb, Mg, Cs, W); (Y, Sr, Ho, Ag, Ce, Te, Pt); (Re, Bi, In); (Pd, Hg, Cr, Er, Nd, K); (Zn, P, Ti, Si); (Ca, Al, Cu, Ba, Zr, Dy); (Tb, Gd, Lu); (V, Au, Rh); (Ta, Ga). In turn, in the study group, there are the following clusters (Figure 3b): (Sr, Cr, Rh, Ti, Mg, Cs, Rb, Be, Sm, Ag, Y, Tl); (Yb, La, W, In); (Ca, Al, Cu, Ba, Pt); (V, Lu, Co); (Nd, K, Si, Sb, Fe); (Ru, Ga, Gd, P, Na, Zr; (Pb, Mo, Ge, Ni, Tb, Zn, Te, Pr); (Tb, Eu); (Os, Dy, Ho). Negative correlations are clearly visible only in the control group and, interestingly, they are completely leveled in the examined group. This applies to the negative correlation of sodium with the following elements: Yb, Hf, La, Tl, Th, Sb, Sm, Be, Rb, Mg, Cs, Ge, Mo, Bi, In, Pt, Te, Ce, Ag, Ho, Y, and potassium with elements such as Mg, Be, Sm, Y.

The correlation matrix for the diabetes control group (Figure 4a) contains the following clusters of strongly positively correlating elements: (Sm, Be, Mg, Tl, Th, Yb, La, Te, Rb, Hf, Mo, Ge, Pb, Tb, W, Sb); (Pd, Hg, Cr, Zr, Er); (Zn, P, Ti—strong); (Y, Sr, Pr, Cs, Ca, Al, Cu, Ba, Pt); (Re, Bi, Ho, Ce, Ag); (Nd, K, Si, Ru). The distinguished clusters in the matrix developed for the study group (Figure 4b) are as follows: (Fe, Cr, Yb, Ag, Tl, Rb, Be, Sm, Y, Pt, Cs, Cu, Al, Sr, Th, Mg, Ti, Rh, Ce); (Os, Dy, Sb); (Ca, Ba); (Pd, Nd, P, K); (W, In); (Pb, Hg, Ru, Na, Gd, Er); (Pr, Te, Mo, Zn, Ga, Zr). Single negative correlations that appear in both the matrix of the test group and the control group are of low significance.

Analyzing the inter-elemental correlations, it should be highlighted that the positive correlations dominate (red fields), whereas negative correlations (blue areas) or their absence (white areas) are much less frequent. Moreover, the clusters formed for the test groups, including patients with additional diseases such as AMD, diabetes, hypertension, and retinopathy, have greater strength in relation to the respective controls.

It is noteworthy that, apart from isolated pairs of elements, negative correlations mainly concerned sodium, and more rarely, potassium and magnesium. This observation appears to be very interesting in the context of metals homeostasis. The membrane transport of alkali (Na^+^, K^+^) and alkali earth (Mg^2+^, Ca^2+^) ions by channels, carriers, and pumps has been studied for many decades. These ions as free or hydrated forms are the most abundant, and the mechanisms of their translocation are well known. It was established that the transport is governed mainly by electrochemical gradients supported with transmembrane protein transporters [32]. During translocation, macrominerals such as hard Lewis acids prefer interactions with hard Lewis bases with oxygen in binding sides, whereas transient metals such as soft Lewis acids interact with soft Lewis base ligands through sulfur or nitrogen in the transmembrane binding sites [33]. However, the metal binding sites are not very selective, due to the flexibility of static prosthetic groups [34], or similarity of metal coordination architectures [35]. It was previously established that binding sites of Cu^+^ transporters can also interact with Ag^+^, Au^+^, Pb^2+^ and Cd^2+^ [35,36]. In turn, Zn^2+^-ATPases are able to bind other metal ions such as Cu^2+^, Co^2+^, and Ni^2+^ [37]. That is why we observed strong cross-correlations in matrices constructed for a large number of elements.

It is significant that the remaining studied elements located mainly in the d- and f-blocks of the periodic table, characterized by the incomplete d- or f-shell, show a negative correlation with macrominerals. This fact can be explained by considering the transmembrane transport rate. In comparison, transport rates of alkali ions change from about 100 ions/s to about 200 ions/s for the Na^+^ or K^+^ channels [38], and ion pumps [39], respectively. In contrast to fast transporting alkali ions, transition ions such as Cu^+^ or Zn^2+^ transport rates are smaller than 10 ions/s [40,41]. Thus, it is alkali/alkali earth metals traveling at diffusion rates that ensure osmotic and electrical balance by mass/charge redistribution. It should be emphasized, however, that AH is not solely a plasma ultrafiltrate, and inter-elemental correlations, and even concentration levels, may be different.

### 2.3. The Statistically Significant Inter-Correlations

The most obtained correlations did not achieve a statistical significance level of *p* < 0.05. However, some elements were significantly inter-correlated. Table 2 only lists the elements exhibiting significant differences between selected patients’ groups in comparison to the appropriate control.

**Figure 5:**
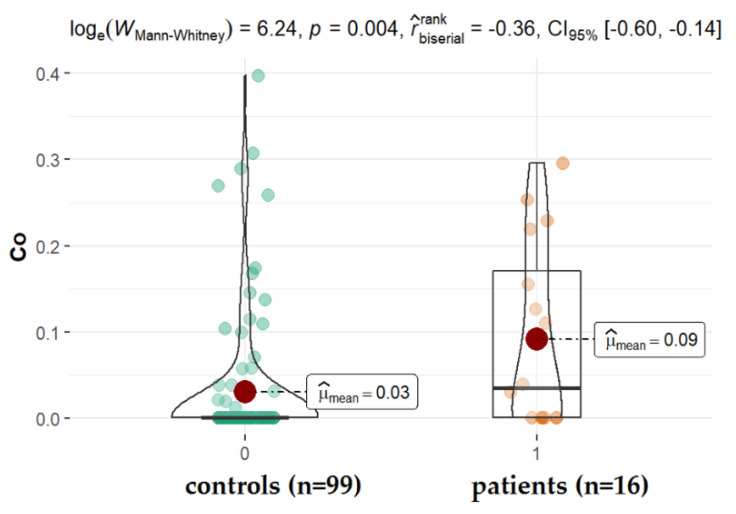
Statistical significance of differences of Co content in AH of patients with AMD compared to control.

AMD is the progressive degeneration of the retinal pigment epithelium (RPE), retina, and choriocapillaris. This disease is observed among elderly people and is the leading cause of irreversible vision loss worldwide [42]. The molecular mechanisms underlying AMD still remain unknown, and the only treatment option for people with AMD is limited to intravitreal injections of anti-VEGF (vascular endothelial growth factor) drugs dedicated for patients with the exudative form. In our study, no significant differences were detected in the aqueous levels of all studied elements between patients with and without AMD (Table 2; *p* > 0.05, Figure 5), except cobalt (Co). Since cobalt is an essential metal and a component of vitamin B12, it is found in most human tissues. Although the toxicity of Co excess has been described, its involvement in AMD pathogenesis still requires more study. It has been proven that Co belongs to a group of 2b IARC carcinogens, it may share an inflammatory pathway, and is also a potent sensitizer [43]. It has been proven that Co causes DNA fragmentation, the activation of caspases, the increased production of ROS (reactive oxygen species), and the secretion of beta-amyloid [29,30,44,45,46,47]. Furthermore, CoCl_2_ exposure coincides with the depression of intracellular Zn^2+^ and Mg^2+^ [30] and hypoxic processes that have been described by Maliha et al. [48]. In our study, patients with AMD had significantly higher levels of cobalt (median: 0.091 ppm, *p* = 0.004) when compared with patients without AMD. This elevated cobalt level in the AH of patients with AMD was also observed previously by others [49]. However, the major limitation of the mentioned study was the small sample size (12 patients). Our finding (16 patients, 99 controls) confirms this observation, suggesting the key role of cobalt in the pathogenesis of AMD. Despite age, which is a major risk factor for AMD, another factor doubling the risk of the problem is smoking. Among the numerous harmful and toxic constituents in tobacco and tobacco smoke, selected metals, such as cobalt, may contribute to the overall harm. This finding, however, requires more evidence and the enrollment of smoking patients in the study.

**Figure 6:**
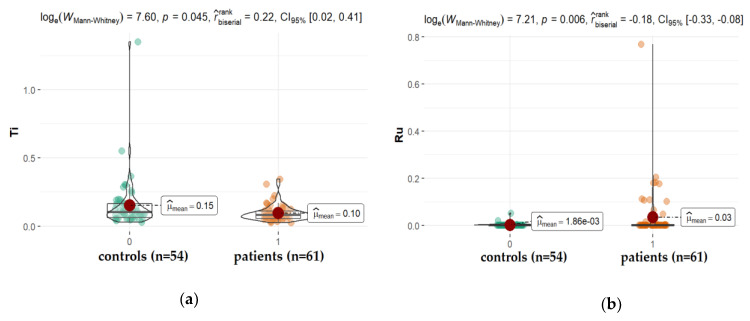

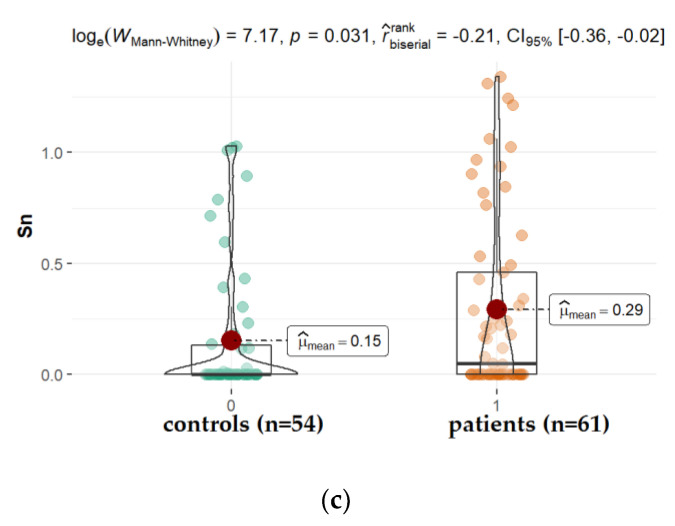
Statistical significance of differences in Tl (**a**), Ru (**b**), and Sn (**c**), content in AH of patients with hypertension compared to controls.

Arterial hypertension is one of the other possible contributors to the etiopathology of cataracts, especially the posterior subcapsular subtype [50,51]. In the present study, we observed significantly higher levels of tin (Sn) and ruthen (Ru), while titanium (Ti) levels (mean: 0.096 ppm, vs. 0.152 ppm for studied and control group, respectively, *p* = 0.045) were reduced in the AH of patients diagnosed with hypertension when compared with patients without this disorder (Figure 6). Ti is a component of alloys used, among others, in medicine for the production of bio-implant materials such as dental prostheses and orthopedic prostheses, but also cosmetics, drugs, confections, and paints [52]. Titanium materials are usually alloyed with aluminum (Al) and vanadium (V). Corrosion products released from the surfaces of dental implants can be swallowed. The absorbed titanium can accumulate in different organs in the body as shown in the animal and cultured cell experiments [53]. Our finding proves that Al, V, Ti appeared in the following order: Al > Ti > V; however, only the level of Ti is significantly higher in diabetes, retinopathy, and AMD in relation to hypertension. Little is known regarding whether metals such as ruthenium (Ru) or tin (Sn) can be deposited in the ocular tissue. Ru-106 is one of the long-lived radionuclides involved in the atmospheric testing of nuclear weapons. In 2017, the Polish Atomic Energy Agency reported Ru-106 detection in the air in Poland. Current indications of national measuring stations confirm the presence of this isotope in the Polish air. Ru-106 is also a radioactive isotope used, among others, in the treatment of cancer. In our study, Ru was found in the AH of all patients; however, patients with hypertension had a significantly higher level of this element in comparison with other groups. In the case of hypertension patients, it is noticeable that Sn concentrations in AH are substantially higher in comparison to controls. Until now, there have been no reports on this in the literature. Therefore, further studies are warranted to corroborate this finding. Sn is not an essential element for humans. This element appears in its inorganic and organic compounds in two oxidation states: +2 and +4. The inorganic tin compounds are non-toxic, whereas the lipid-soluble organic species exhibit toxic reactions. The organotin compounds disturb Ca^2+^ homeostasis. The toxic effect is the result of increasing free intracellular Ca^2+^ concentration, which, among others, affects signaling pathways and induces the apoptosis, depolymerization, and disintegration of proteins. Human exposure to tin arises from the release of the metal and its compounds from natural and anthropogenic sources [54,55,56].

**Figure 7:**
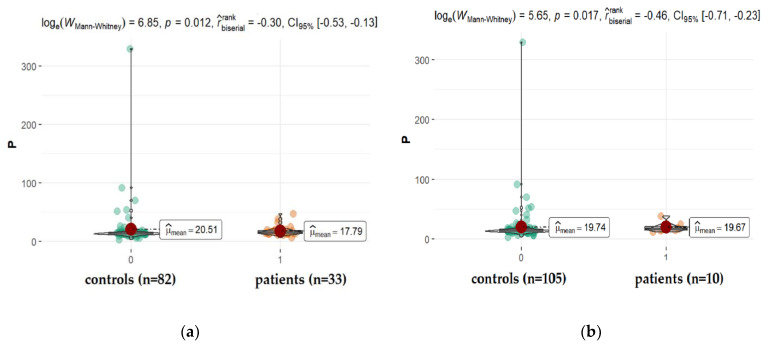
Statistical significance of differences in P content in AH of patients with coexisting diabetes (**a**), and retinopathy (**b**) compared to controls.

The concentration of phosphorous (P) in AH of patients suffering from diabetes and diabetic retinopathy was significantly smaller in comparison to appropriate controls (Figure 7). One might find some information in the literature on the level of phosphorus in the eye fluid. While its concentration is detected at a level, on average around 30 ppm (14.3–43.8 ppm), the changes that appear in the presence of co-morbidities such as diabetes are unclear. Furthermore, sometimes, the observed differences contradict each other. While Kayiklik et al. [26] found no statistically significant differences in the level of P in the eye fluid in diabetic patients compared to those without diabetes, Kim et al. [31] noticed the increase in its content. It is difficult to compare different studies simply by using one parameter measured by various techniques, at varied conditions in samples coming from patients usually suffering from many other disorders such as hypertension, cancer, cardiovascular disorders, or other systemic diseases.

A much better parameter reflecting the metabolism of phosphorus in the body, which eliminates these individual differences, is the measurement of the P/Ca ratio. It was confirmed that the high concentration of serum phosphorus in diabetics is a result of renal insufficiency. When high levels of phosphorus are maintained for long periods, the driving force for mineralization appears and calcium phosphate may be deposited in abnormal sites (ectopic calcification), such as endothelial plaques in the arterial wall prosthetic, valvular, and extraskeletal calcifications [57,58], and even hydrophilic acrylic foldable intraocular lenses opacification, lowering its concentration in body fluids [59,60]. Thus, even if the levels of AH calcium and phosphorous are not statistically different between diabetics and non-diabetics, the ratio P/Ca slightly increases in the diabetes group (*p* = 0.014) similarly to P/Zn (*p* = 0.015).

Without doubt, our study extends the scope of the metallomics research that has been limited to only a few selected elements so far. The strengths of this study also included a large control and study group and the consideration of the most common comorbidities as a variable. However, our study also had its limitations.

First, we were not able to take into account other variables that potentially affect the levels of elements in body fluids, i.e., age, diet, and environmental exposure. Cataracts is a disease that usually appears late in life. Most patients undergoing cataract surgery are over 70 years of age. All patients came from one region of south-eastern Poland, so it can be roughly assumed that they were subjected to similar living conditions.

Secondly, we did not verify other biological fluids or tissues that would allow us to evaluate the distribution of elements more broadly, as such studies were not covered by the approval of the Bioethics Committee.

Thirdly, we had no influence on the possible contamination of the eye fluid during surgery, treatment, or possible supplementation. There was also no data to assess how comorbidities contribute to the progression of cataracts. Such analyses would require the long-term observation of selected groups of patients.

## 3. Materials and Methods

### 3.1. Subjects

The study population included patients attending the Department of General and Pediatric Ophthalmology at the Medical University of Lublin in Poland during the 2019–2021 period. The target population was cataract patients. The inclusion criteria covered the patients posted for cataract surgery in accordance with the applicable diagnostic and procedural codes developed by experts of the Polish Ophthalmology Society on 26 June 2018. Cataract patients diagnosed with additional comorbidities such as non-insulin-dependent diabetes mellitus (diabetes), hypertension, diabetic retinopathy, or age-related macular degeneration (AMD) were selected as cases, while the absence of certain comorbid diseases constituted controls. Patients with comorbidities received routine treatment appropriate to the disease using medicinal products prescribed “on-label” by attending physicians.

The research material consisted of the fluid from the anterior chamber of the eye taken from patients at the beginning of the cataract extraction surgery. The cataract surgery was being performed in only one eye in each patient (thus, numbers of patients = numbers of eyes, within each group). The study was approved in 26 September 2019 by the Local Bioethical Committee of the Medical University of Lublin (approval No. KE-0254/271/2019). The work was carried out in accordance with the Code of Ethics of the World Medical Association (Declaration of Helsinki) for experiments involving humans. The demographic characteristics of the patients are presented in Table 3.

### 3.2. Sample Preparation Procedure

The procedure was described previously [20]. In brief: the samples were stored in 1.5 mL polypropylene tubes at −80 °C until analysis. The wet mineralization of each sample was performed via the addition of 2 mL of 65% suprapur nitric acid HNO_3_ (Merck, Darmstadt, Germany), followed by heating to 180 °C in closed Teflon containers in the microwave mineralization system Mars 6 (CEM, Matthews, NC, USA). Finally, the samples were diluted to 10 mL by ultrapure water obtained in the purification system Milli-Q (Millipore, Darmstadt, Germany).

### 3.3. ICP-OES Measurements

The inductively coupled plasma optical emission spectrometer Agilent 5110 ICP-OES (Agilent, Santa Clara, CA, USA) was employed for elemental analysis. In doing so, the radio frequency (RF) power was 1.2 kW, the nebulizer gas flow was 0.7 L min^−1^, the auxiliary gas flow was 1.0 L min^−1^, the plasma gas flow was 12.0 L min^−1^, the charge-coupled device (CCD) temperature was −40 °C, the viewing height for the radial plasma observation was 8 mm, and the accusation time was 5 s. The analysis was repeated three times. ICP commercial analytical standards (Romil, Cambridge, UK) were used for the calibration. The detection limits (LOD) were determined through 3-sigma criteria and were on the level of 0.00X mg/L (0.001–0.009 mg/L) wet weight (*w/w*) for all elements determined. The uncertainty (determined from the uncertainty budget, coverage factor k = 2 for 95% confidence) for the complete analytical process (including the sample preparation) was at the level of 20%. Due to lack of proper Certified Reference Material, the traceability was assessed by a standard addition procedure for a real sample. A recovery of 80–120% was considered acceptable for all the examined elements.

### 3.4. Statistical Analysis

The inter-elemental correlation analysis was conducted in the studied groups for all elements. All coefficients of Spearman correlation were clustered into homogenous groups within the control and experimental sample with hierarchical cluster analysis. To perform comparisons between groups in the context of element concentration, normality tests (Shapiro–Wilk test) and tests of homogeneity of variance (Levene test) were performed [61,62,63]. The pronounced right-handed asymmetries of the elemental concentration distributions in all the analyzed groups were observed. Due to the fact that both the assumptions of normality and homogeneity of variance were not met in most cases, the Wilcoxon-Mann–Whitney test, being the nonparametric counterpart of the *t*-Student test, was used for intergroup comparisons [64,65].

To assess effect sizes, a biserial rank correlation coefficient was used, which takes values in the range (−1, 1), where (−1) means that values in the first population are smaller than values in the second population [66]. Conversely, if the coefficient is equal to 1, then values in the first population are consistently greater than values in the second population. There are many guidelines of effect size interpretation [67,68,69,70], but the newest [71] suggests considering small effects when r < 0.2, medium effects when r lies in the range of 0.2, 0.3, and when r > 0.3, as large.

All statistical analyses were performed in the R statistical environment (R Core Team 2016) [72], also using a number of libraries that extend the capabilities of the core version of the program. The most important R language libraries used in the analyses are: ggplot2 [73], ggstatsplot [74], rstatix [75], and gtsummary [76].

## 4. Conclusions

In this study, AH, collected from patients undergoing cataract surgery was analyzed by ICP-OES for the presence of 67 elements. The patients had the most common cataract accompanying diseases such as AMD, retinopathy, hypertension, or diabetes. The performed statistical analysis revealed substantial variations in elemental composition between test groups in comparison to the control group with no co-existing morbidity. However, only a relatively small number of elements exhibited statistically significant variation. Levels of P and the ratio of P/Ca were different in retinopathy and diabetes. Levels of Co were significantly altered in AMD. In patients with hypertension, levels of Sn, Ti, and Ru varied significantly from the control group. Knowledge about the inter-correlations between elements in eye fluid may be useful in developing a strategy to combat cataracts, and planned future studies devoted to its pathogenesis.

## Figures and Tables

**Table 1:** Elemental mean concentrations (mg L^−1^) together with the standard deviations (±SD), *p*—probability level, ∆—blank corrected values of AH of studied patients’ groups: c—control group, s—studied group.

Element/Parameter		AMD	Retinopathy	Hypertension	Diabetes
Ca	C	296 ± 167	296 ± 167	304 ± 193	311 ± 178
	S	287 ± 132	276 ± 104	286 ± 131	252 ± 103
	Δ	−8.08	−20.63	−18.10	−59.13
	*p*	0.997	0.988	0.989	0.137
Cs	C	87.6 ± 65.4	90.2 ± 67.1	97.6 ± 72.7	93.0 ± 68.8
	S	103 ± 68	84.4 ± 50.6	82.7 ± 58.5	81.5 ± 57.4
	Δ	14.913	−5.79	−14.92	−11.55
	*p*	0.381	0.893	0.254	0.378
K	C	110 ± 82	108 ± 80	106 ± 86	111 ± 88
	S	103 ± 55	116 ± 72	111 ± 73	103 ± 52
	Δ	−6.63	8.27	5.37	−8.49
	*p*	0.450	0.172	0.339	0.514
Mg	C	16.9 ± 5.1	17.0 ± 5.0	16.9 ± 5.0	17.1 ± 5.4
	S	17.5 ± 5.4	16.1 ± 6.3	17.0 ± 5.2	16.5 ± 4.3
	Δ	0.625	−0.934	0.170	−0.628
	*p*	0.256	0.195	0.900	0.750
Na	C	2180 ± 690	2180 ± 660	2080 ± 400	2170 ± 610
	S	2150 ± 250	2130 ± 400	2260 ± 790	2200 ± 730
	Δ	−28.37	−45.39	184.75	33.46
	*p*	0.837	0.717	0.105	0.924
P	C	20.6 ± 33.7	19.7 ± 32.7	22.8 ± 43.8	20.5 ± 36.8
	S	14.3 ± 2.7	19.7 ± 7.6	17.0 ± 12.4	17.8 ± 8.0
	Δ	−6.29	−0.074	−5.85	−2.72
	*p*	0.824	**0.017**	0.891	**0.012**
Rb	C	14.5 ± 9.6	14.5 ± 8.9	15.4 ± 10.6	14.8 ± 9.6
	S	15.0 ± 6.3	14.6 ± 11.9	13.8 ± 7.7	14.0 ± 8.0
	Δ	0.564	0.016	−1.58	−0.764
	*p*	0.266	0.616	0.664	0.983

**Table 2:** Results of statistically significant differences between studied (s) and control (c) patients’ groups enrolled in the study performed by non-parametric Mann–Whitney U testing.

Cluster/Element		Disease	Mean Conc. (mg L^−1^)	Median (mg L^−1^)	Conc. Range (mg L^−1^)	log_e_ *W*_(MannWhitney)_	R ^1^	CI_95%_^2^	*p* ^3^
I	**Co**	AMD	0.031 (c) 0.091 (s)	0.000 (c) 0.035 (s)	0.000–0.40 0.000–0.30	6.24	−0.36	−0.63; −0.08	0.004
	**Sn**	Hypertension	0.15 (c) 0.29 (s)	0.000 (c) 0.049 (s)	0.000–1.03 0.000–1.34	7.17	−0.21	−0.38; −1.47	0.031
III	**Ru**	Hypertension	0.002 (c) 0.035 (s)	0.000 (c) 0.000 (s)	0.000–0.051 0.000–0.77	7.21	−0.18	−0.28; −0.07	0.006
IV	**Ti**	Hypertension	0.150 (c) 0.096 (s)	0.100 (c) 0.083 (s)	0.029–1.35 0.025–0.34	7.60	0.22	0.04; 0.38	0.045
V	**P**	Retinopathy	19.7 (c) 19.7 (s)	13.9 (c) 17.7 (s)	2.74–329 12.2–38.2	5.65	−0.46	−0.76; −0.09	0.017
	**P**	Diabetes	20.5 (c) 17.8 (s)	13.7 (c) 15.3 (s)	2.74–329 6.66–47.7	6.85	−0.30	−0.49; −0.08	0.012

^1^ r—the rank-biserial correlation; ^2^ CI—a confidence interval; ^3^ *p*—the probability value (*p*-value) in the Mann–Whitney U test.

**Table 3:** Demographic characteristics of the patient groups enrolled in the study.

Disease	Group	Gender	*n*	%	Min–Max Age	Median Age	Mean Age ± SD
**AMD**	studied	Female	11	68.75	67–88	76.0	76.18 ± 6.24
		Male	5	31.25	74–89	81.0	80.80 ± 6.06
	control	Female	63	63.64	55–94	76.0	75.46 ± 7.13
		Male	36	36.36	58–89	72.5	72.83 ± 7.50
**Retino-pathy**	studied	Female	5	50.00	69–86	77.0	76.80 ± 6.10
		Male	5	50.00	67–77	71.0	72.00 ± 4.00
	control	Female	69	65.71	55–94	76.0	75.48 ± 7.06
		Male	36	34.29	58–89	74.5	74.06 ± 8.13
**Hyper-tension**	studied	Female	42	68.85	60–94	76.0	75.81 ± 6.26
		Male	19	31.15	63–89	74.0	75.21 ± 7.35
	control	Female	32	59.26	55–91	77.0	75.25 ± 7.91
		Male	22	40.74	58–84	75.0	72.59 ± 8.02
**Dia-betes**	studied	Female	18	54.55	55–86	74.5	73.50 ± 7.48
		Male	15	45.45	63–81	74.0	73.60 ± 5.68
	control	Female	56	68.29	59–94	76.5	76.23 ± 6.74
		Male	26	31.71	58–89	74.5	73.92 ± 8.81

## Data Availability

The data presented in this study are available on request from Joanna Dolar-Szczasny.

## Supplementary Materials

The following are available online at https://www.mdpi.com/article/10.3390/ijms22179413/s1.
