# Supplementary material for: The Multi-Elemental Composition of the Aqueous Humor of Patients Undergoing Cataract Surgery, Suffering from Coexisting Diabetes, Hypertension, or Diabetic Retinopathy
Source: Int J Mol Sci. 2021 Aug 30;22(17):9413. doi: 10.3390/ijms22179413 (PMC8430749; doi:10.3390/ijms22179413)
Supplement: Supplementary file 1 [file ijms-22-09413-s001.zip › ijms-1281342-supplementary.pdf]

Table S1 Mean values of elemental concentration (ppm) in the aqueous humor of cataract patients suffering additionally from AMD, diabetes, retinopathy, hypertension alongside with standard deviation (SD). Division of elements into clusters according to Ref. 21.

		AMD		Retinopathy		Hypertension		Diabetes	
		Mean [ppm]	SD	Mean [ppm]	SD	Mean [ppm]	SD	Mean [ppm]	SD
**Cluster 1**									
**Ag**	C	0.014	0.028	0.014	0.028	0.018	0.032	0.015	0.03
	S	0.016	0.021	0.01	0.021	0.011	0.022	0.012	0.018
**Ce**	C	0.025	0.056	0.023	0.054	0.028	0.071	0.025	0.058
	S	0.016	0.025	0.023	0.04	0.019	0.028	0.02	0.034
**Co**	C	0.031	0.075	0.041	0.085	0.055	0.097	0.04	0.087
	S	0.091	0.107	0.018	0.044	0.025	0.064	0.036	0.071
**Cr**	C	0.043	0.122	0.032	0.044	0.036	0.052	0.034	0.046
	S	0.03	0.035	0.137	0.364	0.045	0.15	0.059	0.202
**Ga**	C	0.067	0.136	0.071	0.138	0.073	0.146	0.074	0.136
	S	0.077	0.118	0.045	0.066	0.065	0.122	0.054	0.125
**Hg**	C	0.199	0.486	0.2	0.473	0.248	0.628	0.212	0.524
	S	0.138	0.182	0.084	0.164	0.139	0.202	0.136	0.201
**Ho**	C	0.021	0.053	0.023	0.053	0.028	0.068	0.023	0.057
	S	0.025	0.04	0.001	0.002	0.016	0.029	0.018	0.031
**Pb**	C	0.583	1.309	0.618	1.384	0.615	1.206	0.638	1.483
	S	0.812	1.665	0.586	1.105	0.616	1.489	0.559	0.997
**Pr**	C	0.021	0.037	0.019	0.036	0.019	0.029	0.02	0.038
	S	0.006	0.012	0.012	0.016	0.018	0.04	0.014	0.025
**Rh**	C	0.061	0.083	0.06	0.076	0.064	0.079	0.057	0.076
	S	0.064	0.084	0.077	0.143	0.059	0.086	0.074	0.098
**Sb**	C	0.365	0.762	0.363	0.748	0.392	0.868	0.382	0.801
	S	0.191	0.338	0.106	0.169	0.296	0.56	0.238	0.452
**Si**	C	1.793	4.02	2.057	4.863	2.204	4.643	2.22	5.058
	S	3.183	7.732	1.248	2.168	1.793	4.761	1.406	3.62
**Sn**	C	0.249	0.382	0.224	0.364	0.152	0.3	0.263	0.398
	S	0.089	0.227	0.262	0.426	0.293	0.409	0.137	0.263
**Ta**	C	0.075	0.148	0.076	0.156	0.083	0.191	0.076	0.165
	S	0.056	0.168	0.029	0.042	0.062	0.102	0.063	0.105
**Yb**	C	0.006	0.006	0.006	0.006	0.006	0.006	0.007	0.006
	S	0.008	0.006	0.006	0.008	0.006	0.006	0.006	0.006
**Zr**	C	0.019	0.025	0.02	0.026	0.022	0.031	0.021	0.027
	S	0.023	0.023	0.017	0.019	0.017	0.019	0.016	0.018
**Cluster 2**									
**Al**	C	2.43	1.742	2.444	1.722	2.627	2.064	2.592	1.856
	S	2.404	1.256	2.247	1.171	2.249	1.233	2.016	1.032
**Ba**	C	3.651	4.69	3.649	4.699	3.888	5.143	4.068	5.065
	S	2.908	3.628	2.483	2.401	3.246	3.973	2.253	2.534
**Fe**	C	1.816	4.985	1.634	4.633	1.342	2.822	1.788	5.162
	S	0.924	1.089	2.306	5.031	2.002	5.816	1.455	3.08
**In**	C	1.126	1.164	1.093	1.142	1.045	0.942	1.186	1.199
	S	1.077	0.991	1.396	1.117	1.185	1.291	0.955	0.967
**Pt**	C	1.333	1.18	1.404	1.314	1.431	1.345	1.462	1.403
	S	1.94	1.809	1.564	1.099	1.406	1.257	1.307	0.979
**Sm**	C	0.833	0.709	0.836	0.69	0.928	0.803	0.852	0.75
	S	0.884	0.536	0.879	0.668	0.762	0.557	0.811	0.5
**Te**	C	2.633	1.85	2.69	1.819	2.903	2.013	2.819	1.902
	S	2.914	1.705	2.493	1.985	2.468	1.632	2.309	1.589
**Tl**	C	3.07	2.816	3.121	2.804	2.812	2.806	2.876	2.7
	S	3.295	2.856	2.892	3.02	3.357	2.812	3.659	3.038
**W**	C	1.162	1.629	1.19	1.665	1.311	2.028	1.265	1.842
	S	1.059	1.475	0.704	0.517	1.003	1.096	0.857	0.66

Table S1 Mean values of elemental concentration (ppm) in the aqueous humor of cataract patients suffering additionally from AMD, diabetes, retinopathy, hypertension alongside with standard deviation (SD). Division of elements into clusters according to Ref. 21.

**Zn**	C	0.37	0.554	0.364	0.54	0.44	0.719	0.395	0.602
	S	0.278	0.198	0.281	0.203	0.284	0.212	0.262	0.183
**Cluster 3**									
**As**	C	0.076	0.333	0.097	0.431	0.094	0.391	0.068	0.316
	S	0.221	0.769	0.091	0.287	0.099	0.446	0.168	0.605
**B**	C	0	0	0	0	0	0	0	0
	S	0	0	0	0	0	0	0	0
**Cd**	C	0.002	0.012	0.002	0.012	0.002	0.01	0.003	0.013
	S	0	0	0.002	0.003	0.002	0.012	0.001	0.002
**Dy**	C	0.007	0.012	0.007	0.011	0.007	0.012	0.006	0.011
	S	0.003	0.008	0.004	0.006	0.006	0.01	0.008	0.012
**Er**	C	0.003	0.008	0.003	0.008	0.003	0.007	0.003	0.008
	S	0.004	0.009	0.001	0	0.003	0.009	0.002	0.007
**Eu**	C	0.001	0.002	0.001	0.002	0.001	0.003	0.001	0.002
	S	0.001	0.001	0.001	0	0.001	0.001	0.001	0.001
**Gd**	C	0.003	0.007	0.003	0.009	0.002	0.006	0.003	0.008
	S	0.005	0.014	0	0	0.004	0.01	0.004	0.01
**Li**	C	0.019	0.185	0.018	0.179	0.035	0.25	0.023	0.203
	S	0.001	0.003	0	0	0.001	0.001	0.001	0.002
**Lu**	C	0.003	0.008	0.003	0.008	0.003	0.005	0.003	0.008
	S	0.002	0.005	0.001	0.002	0.003	0.009	0.003	0.007
**Mn**	C	0.001	0.004	0.001	0.001	0.001	0.001	0.001	0.001
	S	0	0	0.004	0.011	0.001	0.005	0.002	0.006
**Nd**	C	0.007	0.021	0.007	0.02	0.007	0.019	0.009	0.023
	S	0.007	0.016	0.007	0.02	0.008	0.022	0.003	0.012
**Os**	C	0.018	0.068	0.013	0.053	0.015	0.061	0.008	0.025
	S	0.009	0.02	0.053	0.131	0.019	0.066	0.039	0.11
**Pd**	C	0.019	0.093	0.017	0.09	0.027	0.123	0.019	0.101
	S	0.005	0.02	0.017	0.041	0.008	0.024	0.011	0.026
**Ru**	C	0.02	0.085	0.021	0.084	0.002	0.007	0.016	0.045
	S	0.013	0.051	0.004	0.007	0.035	0.109	0.028	0.134
**Sc**	C	0.002	0.012	0.002	0.012	0.004	0.016	0.003	0.013
	S	0.001	0.001	0	0	0.001	0.003	0	0
**Tb**	C	0.007	0.017	0.008	0.018	0.009	0.021	0.006	0.014
	S	0.01	0.017	0.006	0.009	0.006	0.013	0.011	0.023
**Tm**	C	0.003	0.019	0.003	0.018	0.002	0.006	0.001	0.005
	S	0	0	0	0	0.003	0.023	0.006	0.031
**U**	C	0.007	0.026	0.007	0.025	0.009	0.031	0.004	0.017
	S	0	0	0	0	0.003	0.016	0.011	0.037
**V**	C	0.006	0.019	0.008	0.024	0.007	0.024	0.007	0.022
	S	0.014	0.043	0.004	0.011	0.008	0.023	0.008	0.027
**Cluster 4**									
**Au**	C	1.423	2.147	1.646	2.698	1.455	2.672	1.731	2.879
	S	3.111	4.497	1.785	2.027	1.838	2.62	1.477	1.946
**Be**	C	0.038	0.031	0.038	0.028	0.04	0.033	0.038	0.03
	S	0.041	0.021	0.041	0.046	0.037	0.026	0.039	0.029
**Bi**	C	0.272	0.403	0.284	0.411	0.247	0.468	0.278	0.429
	S	0.297	0.422	0.184	0.333	0.301	0.341	0.268	0.342
**Cu**	C	0.155	0.135	0.152	0.129	0.174	0.162	0.163	0.141
	S	0.146	0.074	0.173	0.12	0.137	0.085	0.132	0.084
**Ge**	C	0.46	0.968	0.501	0.994	0.667	1.212	0.545	1.082
	S	0.586	0.909	0.224	0.318	0.309	0.618	0.309	0.507
**Hf**	C	0.224	0.206	0.233	0.209	0.244	0.229	0.232	0.214
	S	0.221	0.204	0.119	0.108	0.205	0.18	0.202	0.181
**Ir**	C	0.692	1.05	0.74	1.127	0.731	1.062	0.648	1.016
	S	0.942	1.39	0.59	0.782	0.723	1.141	0.922	1.281

Table S1 Mean values of elemental concentration (ppm) in the aqueous humor of cataract patients suffering additionally from AMD, diabetes, retinopathy, hypertension alongside with standard deviation (SD). Division of elements into clusters according to Ref. 21.

**La**	C	0.019	0.012	0.019	0.012	0.019	0.013	0.019	0.013
	S	0.018	0.014	0.016	0.014	0.018	0.012	0.017	0.01
**Mo**	C	0.287	0.373	0.282	0.359	0.283	0.362	0.289	0.376
	S	0.262	0.284	0.295	0.403	0.283	0.363	0.27	0.326
**Ni**	C	0.263	0.422	0.247	0.406	0.266	0.424	0.271	0.445
	S	0.167	0.279	0.281	0.427	0.235	0.392	0.197	0.285
**Re**	C	0.491	0.726	0.521	0.732	0.595	0.874	0.531	0.776
	S	0.679	0.591	0.474	0.423	0.448	0.521	0.483	0.516
**Se**	C	0.6	0.695	0.619	0.706	0.625	0.716	0.629	0.662
	S	0.671	0.728	0.514	0.617	0.596	0.684	0.561	0.785
**Sr**	C	0.118	0.073	0.12	0.069	0.115	0.072	0.119	0.07
	S	0.145	0.09	0.138	0.13	0.128	0.079	0.129	0.09
**Th**	C	0.138	0.205	0.135	0.2	0.171	0.244	0.149	0.218
	S	0.131	0.154	0.155	0.185	0.107	0.141	0.108	0.134
**Ti**	C	0.125	0.15	0.124	0.144	0.152	0.192	0.133	0.161
	S	0.103	0.05	0.104	0.091	0.096	0.059	0.096	0.056
**Y**	C	0.024	0.023	0.023	0.022	0.024	0.021	0.022	0.02
	S	0.023	0.022	0.026	0.029	0.023	0.024	0.029	0.028
**Cluster 5**									
**Ca**	C	295.632	167.296	296.302	167.057	304.108	192.728	311.475	178.514
	S	287.552	131.729	275.669	104.187	286.009	130.767	252.346	103.153
**Cs**	C	87.667	65.41	90.246	67.126	97.657	72.747	93.055	68.805
	S	102.58	68.146	84.451	50.658	82.734	58.474	81.509	57.395
**K**	C	109.733	82.529	108.091	80.008	105.962	85.628	111.246	87.838
	S	103.103	55.005	116.364	72.345	111.331	73.489	102.756	52.085
**Mg**	C	16.872	5.089	17.04	5.013	16.869	5.072	17.139	5.411
	S	17.497	5.412	16.106	6.329	17.039	5.193	16.511	4.336
**Na**	C	2180.397	687.062	2180.397	663.405	2078.450	401.671	2166.849	607.699
	S	2152.029	251.96	2135.009	399.274	2263.204	792.632	2200.307	734.957
**P**	C	20.609	33.698	19.74	32.731	22.836	43.796	20.514	36.801
	S	14.317	2.726	19.666	7.6	16.987	12.451	17.794	8.032
**Rb**	C	14.473	9.571	14.55	8.931	15.39	10.598	14.77	9.619
	S	15.037	6.323	14.566	11.953	13.808	7.698	14.006	8.048
